# Modern extraction techniques and their impact on the pharmacological profile of *Serenoa repens* extracts for the treatment of lower urinary tract symptoms

**DOI:** 10.1186/1471-2490-14-63

**Published:** 2014-08-11

**Authors:** Celeste De Monte, Simone Carradori, Arianna Granese, Giovanni Battista Di Pierro, Costantino Leonardo, Cosimo De Nunzio

**Affiliations:** 1 Department of Drug Chemistry and Technologies, Sapienza University of Rome, P.le A. Moro 5, Rome 00185, Italy; 2 Department of Obstetrics, Gynecology and Urology, Sapienza University of Rome, Viale del Policlinico 155, Rome 00161, Italy; 3 Department of Urology, Ospedale Sant’Andrea, Sapienza University of Rome, Via di Grottarossa 1035/1039, Rome 00189, Italy

**Keywords:** Benign prostatic hyperplasia, *Serenoa repens*, Extraction techniques, Supercritical fluid extraction, Lipidosterolic composition, Content standardization

## Abstract

**Background:**

Bioactive compounds from plants (*i.e.*, *Serenoa repens*) are often used in medicine in the treatment of several pathologies, among which benign prostatic hyperplasia (BPH) associated to lower urinary tract symptoms (LUTS).

**Discussion:**

There are different techniques of extraction, also used in combination, with the aim of enhancing the amount of the target molecules, gaining time and reducing waste of solvents. However, the qualitative and quantitative composition of the bioactives depends on the extractive process, and so the brands of the recovered products from the same plant are different in terms of clinical efficacy (no product interchangeability among different commercial brands).

**Summary:**

In this review, we report on several and recent extraction techniques and their impact on the composition/biological activity of *S. repens*-based available products.

## Background

Benign prostatic hyperplasia (BPH) is a significant health concern and increases in prevalence as the population ages. Symptomatic BPH represents the most common urologic disease among elderly males, affecting about one-quarter of men in their ‘50s, one-third of men in their ‘60s, and about half of octogenarians. BPH is considered a chronic disease with early initiation and slow progression. BPH starts as a simple micro nodular hyperplasia and evolves into a macroscopic nodular enlargement that may result in bladder prostatic obstruction (BPO), causing lower urinary tract symptoms (LUTS) [1,2]. When considering options for managing LUTS, which may suggest BPO, the short term goal is to improve the individual’s quality of life by relieving symptoms, however, the aim should be to prevent or reduce the worsening of symptoms over longer term and limiting BPH progression [3]. Medical treatment options of LUTS/BPO include α1-adrenoceptor antagonists, 5α-reductase inhibitors, anti-cholinergic agents, phosphodiesterase 5 inhibitors and plant extracts. Although the European Association of Urology is unable to make specific recommendations about plant extracts treatment in patients with LUTS/BPH because of the heterogeneity of the marketed products and the methodological problems associated with meta-analysis, their use in clinical practice is rising with an increased global prescription index, particularly in some European countries (Belgium, Hungary, Poland, France) [2,4,5]. Furthermore, the treatment of BPH with α-blockers and 5α-reductase inhibitors could play an important role in the alteration of sexual functions leading to ejaculatory and erectile disorders [6]. To avoid this issue, natural products derived from plants have been using for treating BPH, especially extracts of *Serenoa repens* (saw palmetto) obtained from the American dwarf palm [7-9].

Nowadays, a lot of extractive strategies with differences in terms of methodology, time, temperature, pressure and solvents have been developed, also used in combination with other techniques in order to improve the recovery and, consequently, the pharmacological profile of their extracts. However, as a consequence of the differences among the extractive processes used by several companies, there is a discrepancy in the qualitative and quantitative composition of the extracts obtained from the same plant. Hence, each brand is different in quality and efficacy for the content of the bioactive compounds with repercussions on the beneficial effects for patients in response to clinical trials and treatments [10].

## Discussion

The aim of this review is the evaluation of the available evidence on plant extraction and its possible clinical implications on *S. repens* therapeutic efficacy.

### *Serenoa repens* as a standard reference material (SRM)

The knowledge of the exhaustive quali-quantitative composition of *S. repens* extracts is warranted not only due to the multiple variables associated to different extraction techniques, but also due to the not yet fully understood pharmacological profile of each active principle.

For this reason, the validation of appropriate analytical methods, to develop standard reference materials for selected dietary supplements, have been regulated by the National Institute of Health’s Office of Dietary Supplements and the Food and Drug Administration’s Center for Drug Evaluation and Research in collaboration with the National Institute of Standards and Technology (NIST) [11].

NIST, as a non-regulatory federal agency of USA, supports accurate and compatible measurements by certifying and providing over 1300 Standard Reference Materials® (SRMs) with well-characterized composition or properties. Each NIST Standard Reference Material® is supplied with a Certificate of Analysis and/or a Materials Safety Data Sheet. In addition, NIST has published many articles and practice guides that describe the development, analysis, traceability and use of SRMs. Certified concentration values are usually determined by two or more independent methods, which could be combined with data from other laboratories.

In the case of saw palmetto, two reference materials have been reported, SRM 3250 *S. repens* ground fruit and SRM 3251 *S. repens* extract. SRM 3250 has certified concentration values for specific phytosterols, and fatty acids (free or as triglycerides). On the other hand, the extract SRM 3251 has certified concentration values for phytosterols, fatty acids (free or as triglycerides), β-carotene and its isomers, and γ- and δ-tocopherol.

For SRM 3250 three extraction procedures and conditions for each procedure were evaluated including PFE (pressurized fluid extraction), Soxhlet extraction (with dichloromethane), and sonication. PFE and Soxhlet extraction gave the highest amounts of extracted fatty acids from the ground fruit. In the case of PFE (conditions: solvent mixture of 4:1 (v/v) of hexane/acetone with a four static cycle extraction at 125°C and 10.4 MPa), the choice of solvent, temperature of extraction, and pressure of extraction did not have significant effects on the composition of the extracted fractions. The solvent choice also did not have a deep impact on the efficiency of the Soxhlet extraction, although the duration of the extraction time was critical (at least 40 h). Conversely, SRM 3251 has been obtained as a supercritical CO_2_ extract and analyzed with respect to the corresponding extracts obtained for SRM 3250.

As reported in Table 1, the concentrations of the fatty acids as triglycerides were 6–25 times higher in SRM 3251 compared with SRM 3250. In general, the concentration of each fatty acid as a triglyceride was higher than the corresponding free fatty acid for both extracts. The concentration of linoleic and α-linoleic acid in SRM 3250 was approximately six times lower than in SRM 3251. Linoleic and α-linolenic acids were also lower in concentration as free fatty acids in both SRM 3250 and SRM 3251 as compared with the corresponding fatty acids as triglycerides.

**Table 1:** **Composition of *Serenoa repens *** **extracts as standard reference materials**

**Extract**	**Fatty acids content (as triglycerides)**	**Free fatty acids content on a dry mass basis**
**SRM 3250 (ground fruit)**	2.96% (dodecanoic acid)	From 0.0121 mg/g (pentadecanoic acid) to 33.7 mg/g (oleic acid)
	3.24% (oleic acid)	
	1.10% (myristic acid)	
	0.869% (palmitic acid)	
	0.824% (linoleic acid)	
	0.194% (linolenic acid)	
	0.179% (stearic acid)	
	0.117% (capric acid)	
	0.107% (caprylic acid)	
	0.0173% (gondoic acid)	
	0.0158% (palmitoleic acid)	
	0.0107% (tetracosanoic acid)	
	0.0097% (arachidic acid)	
	0.0076% (tridecanoic acid)	
	0.0066% (docosanoic acid)	
	0.0061% (heptadecanoic acid)	
	0.0547% (vaccenic acid)	
	0.0047% (pentadecanoic acid)	
**SRM 3251 (supercritical CO**_**2**_**extract)**	34.73% (oleic acid)	From 0.119 mg/g (pentadecanoic acid) to 221 mg/g (oleic acid)
	26.34% (dodecanoic acid)	
	10.62% (myristic acid)	
	8.51% (palmitic acid)	
	5.99% (linoleic acid)	
	2.67% (capric acid)	
	2.65% (caprylic acid)	
	2.25% (hexanoic acid)	
	1.24% (linolenic acid)	
	0.830% (vaccenic acid)	
	0.271% (palmitoleic acid)	
	0.1931% (gondoic acid)	
	0.175% (stearic acid)	
	0.0932% (arachidic acid)	
	0.0926% (tetracosanoic acid)	
	0.069% (tridecanoic acid)	
	0.0644% (docosanoic acid)	
	0.0637% (heptadecanoic acid)	
	0.0515% (pentadecanoic acid)	
	0.0313% (undecanoic acid)	

These SRMs could also furnish appropriate reference materials to make comparisons among dietary supplements. In fact, SRM 3251 has the second highest concentration of linoleic acid, as regards the triglycerides, compared with the other available SRMs (*i.e.*, fish oil and tissues), and the highest concentration of α-linolenic acid. As regards the phytosterols content, in both SRMs, campesterol, β-sitosterol and stigmasterol have certified concentration values (higher in SRM 3251 than SRM 3250). Furthermore, β-carotene (46.8 μg/g), γ-tocopherol (280 μg/g) and δ-tocopherol (35.3 μg/g) were also quantified.

### Extraction techniques

Many plants possess bioactive metabolites that can be used as therapeutic agents for the treatment of human pathologies like BHP associated to LUTS. Because of the very low concentrations of therapeutic compounds in plants, their exhaustive recovery becomes a crucial issue in order to obtain high yields of the products with the use of an extractive method that should be reproducible, time saving and eco-friendly. Furthermore, in the choice of the extractive method, it is important to keep in mind the thermolability of the active molecules, hence the temperature and the other parameters should be optimized. For having an efficient process, three important factors must be mainly considered: the sample matrix, the type and the localization of bioactive compounds within the matrix. In fact, the target compound firstly must be removed from the site in which it is placed and then it must diffuse towards the extraction phase to be finally collected.

We briefly report on classical and innovative extraction techniques which have been or could be applied to obtain *S. repens* extracts enriched by specific active principles.

#### Solvent extraction

According to the solubility of the bioactive compounds there are a large number of inorganic, organic, polar and non-polar solvents to perform a good extraction, also in combination among them. If the substance of our interest is lipophilic, the organic solvents of choice will be non-polar, ranging from those with a very low polarity such as hexane, to those that are less non-polar like chloroform and dichloromethane. For example, the apolar solvents cyclohexane, hexane, toluene, benzene, ether, chloroform and ethyl acetate are currently used to extract alkaloids, coumarins, fatty acids (FAs), flavonoids and terpenoids. On the contrary, for hydrophilic compounds the choice will fall on a polar solvent which may be non-protic such as acetone, or protic such as ethanol, methanol or even water. In fact acetone, acetonitrile, butanol, propanol and ethanol are the solvents for the extraction of flavonols, lectins, alkaloids, quassinoids, flavones, polyphenols, tannins and saponins. One of the major pros of this procedure is the use of simple equipment and its limited cost.

#### Microwave-assisted extraction

Microwave-assisted extraction (MAE) is an extractive method based on the utilization of microwave energy that is produced when the perpendicular oscillation between the electric and the magnetic fields generates electromagnetic radiations with a frequency ranging from 0.3 to 300 GHz. If the microwave goes through and interacts with a substance there is a production of heat whose intensity depends on the absorption of the energy by the material and the dissipation of the resulting heat [12].

MAE techniques can be classified according to the pressure through which they operate: higher than the atmospheric pressure (closed MAE system) and lower than the atmospheric pressure (open MAE system). As regards closed systems, the temperature is set over the boiling point of the solvent and the pressure is under control to avoid an excessive development. Among the closed systems we can enumerate high pressure microwave-assisted extraction (HPMAE) which uses high pressure and temperature in order to enhance the capacity of the solvent to incorporate the energy from radiation and to avoid large amount of solvent for the extraction. In case of thermolabile molecules, soft conditions are needed and so the choice will fall on an open system or the vacuum microwave assisted extraction (VMAE) that allows the reduction of the boiling point of the solvent. For compounds that are susceptible of oxidation it has been developed the nitrogen-protected microwave-assisted extraction operating under pressurized inert gas. When the bioactive compounds are susceptible of hydrolysis such as the essential oils, solvent-free microwave-assisted extraction (SFME) is used to avoid the loss/degradation of these products. Moreover, MAE can be associated to ultrasonic energy (ultrasonic/microwave-assisted extraction UMAE) to reduce extraction times and the amounts of solvent leading to an improvement of the yield. Another way to gain time is coupling the extraction step with the analytical one in the dynamic microwave-assisted extraction (DMAE) which operates continuously and automatically. The choice of the solvent is influenced by its ability to absorb the microwave radiation: ethanol, methanol, water and the more selective room temperature ionic liquids are good solvents for MAE. The ratio of solvent to solid is important for improving MAE: if the amount of solvent is too much it absorbs all the energy with a resulting inefficient matrix heating, whereas if the ratio is too low the amount of solvent is not enough to allow the diffusion of the compounds out of the matrix. Also the vessel size is a critical factor because in a little vessel the internal pressure tends to augment and this could mean a degradation of the more delicate molecules.

Another parameter to consider in MAE is the power of extraction: an increased power boosts the temperature reducing the solvent viscosity and leading to a better efficiency, except in case of thermolabile molecules. Microwave-assisted extraction gives several advantages with respect to classical extractive processes such as Soxhlet: MAE allows a gain of time, higher quality and yields [13]. It is also cheaper than supercritical fluid extraction (SFE) and faster than ultrasonic-assisted extraction (UAE). On the other hand, MAE shows some drawbacks: it is more expensive than UAE, less eco-friendly than SFE due to the use of organic solvents, not suitable for thermolabile compounds because the irradiation could promote chemical reactions with the loss of the desirable products, and not efficient when the target molecules and/or the solvent of extraction are non-polar because they do not absorb energy from the source [14]. This technique has never been applied to *S. repens* extraction.

#### Ultrasound-assisted extraction

In recent times, ultrasound-assisted extraction has received a great interest to overcome the disadvantages of classical solvent extractions such as little yields and waste of time. UAE is based on the production of ultrasound waves and their transmission throughout the solvent with a resulting cavitation. When the cavitation bubbles collapse, there is a generation of liquid circulation currents and turbulence that improve the mass transfer rate. The fractures formed in the cell wall enhance its permeability and so a bigger amount of solvent can enter into the plant tissues to extract the bioactive metabolites. In order to perform an extraction based on sonochemistry, the choice of solvent becomes an important parameter because its physical properties like polarity, viscosity, vapour pressure and surface tension influence the cavitation phenomena. Ethanol, methanol and hexane are very used in UAE, and sometimes water could be added to ethanol, even if its amount must not be too much in order to avoid a decrease in extraction efficiency, probably due to the generation of radicals from the ultrasonic dissociation of water [15]. Other parameters to be considered are the frequency and the power: often the former ranges from 20 to 100 kHz and the latter from 100 to 800 W. Also the power dissipation is a critical factor, because the generation of physical effects like turbulence is directly proportioned to the power dissipated as heat. For the future, the design of reactors based on multiple transducers is needed in order to operate at multiple frequencies and improve the efficacy of UAE. Another problem that currently limits the use of UAE at large scales is the erosion of transducers and their continuous replacement to avoid a decrease in the transmitted energy. Nonetheless, UAE is less expensive than the traditional extractive techniques; it can give high quantities of products without spending time and without using large amounts of solvent. For a better performance, UAE can be also used in combination with other techniques like supercritical fluid process. This technique has never been applied to *S. repens* extraction.

#### Supercritical fluid extraction

Supercritical fluid extraction is a novel technique especially used for the recovery of essential oil from plants. SFE is based on the use of carbon dioxide in supercritical phase, which is at low pressure and temperature (74 bar and 32°C): in this state CO_2_ possesses a polarity similar to pentane and so it is a good candidate for the extraction of lipophilic compounds. Furthermore supercritical CO_2_ is non toxic, non-flammable, not expensive, and easy to remove in the end of the process (eco-friendly). Operating at low temperature it is possible to obtain in high yield thermolabile compounds like terpenes and terpenoids that normally have their boiling point over 150°C and so it is important to work at lower temperatures for preventing their degradation [16]. If the components to extract are polar, a cosolvent like water or ethanol in little percentage (5-10%) is needed to increase the extraction quality. When in the plant matrix there are bioactive compounds of different solubility, a method to improve the recovery of all the phytotherapeutics without any loss is the fractionation of the extract [17]. Two strategies could be applied: the multi-step fractionation and the on-line fractionation.

When the multi-step fractionation is performed, there are different successive steps of separation with different conditions in terms of density of CO_2_: in the first step we will obtain the fraction of the more soluble compounds such as essential oils, whereas in the second step, increasing CO_2_ density we will have the recovery of the less soluble components like antioxidants. Moreover, thanks to this strategy we can obtain in the first step the products extracted by the only use of supercritical CO_2_, while in a second time we can add a cosolvent such as ethanol for the other compounds. The on-line fractionation works, instead, in a cascade depressurization system in order to obtain the precipitation of the several fractions according to their saturation conditions. The particular properties of the supercritical fluid characterized by a low viscosity and a high diffusion make this technique an excellent alternative to the others for the recovery of therapeutic products from plants.

#### Ionic liquids

The use of ionic liquid (IL) for analytical purposes has been developed in modern times with advantages in terms of quality and efficacy of extraction. In particular, an ionic liquid consists of a liquid organic salt that selectively interacts with specific polar and non-polar compounds thanks to ion-exchanges, π-stacking interactions, hydrophobic interactions or hydrogen bonds, improving the selectivity of the extractive method [18]. For example, the interaction of 1-butyl-3-methylimidazolium hexafluorophosphate ([BMIM][PF_6_]) with hydrophilic amino acids enhances the extraction efficiency with respect to a traditional organic solvent extraction. Being ILs applied in several extractive processes such as MAE and UAE, they should be stable enough to resist to the high temperatures through which the technique works. About this matter, tetraalkylammonium cations and imidazolium ions like tetrafluoroborate [BF_4_]^−^, bis(trifluoromethylsulfonyl)imide [NTf_2_]^−^, trifluoromethanesulfonate [CF_3_SO_3_]^−^, and hexafluorophosphate [PF_6_]^−^, possess a great thermal stability; moreover, the organic anions have shown to be more stable than the inorganic ones when operating at elevated temperatures. In conclusion, extraction based on ILs represents a good choice to recover in high yields organic and inorganic and metal ions as bioactive components of plants and herbs. This technique has never been applied to *S. repens* extraction.

#### Enzyme-assisted extraction

An alternative approach to classical solvent extraction techniques is the enzyme-assisted extraction: this method is innovative and convenient thanks to the fact that the enzymes catalyze reactions in a specific way without operating under strong conditions that could lead to the degradation of the desired products. In addition, proteins like cellulases, hemicellulases and pectinases disrupt cell wall with the hydrolysis of its components leading to a major permeability and allowing an easier release of the metabolites from plants [19]. The application of enzymes such as lipases, proteases, phospholipases, permits to reduce the use of the solvent for the extraction. For oil extraction from plants, cellulase, α-amylase and pectinase are the most used enzymes. These proteins can be obtained from fungi, bacteria, animals, and vegetables or from genetic engineering methods and, thanks to their selective catalysis, they can be used to recover a specific bioactive compound in high yields and in a “green” approach, without wasting too much energy. Nevertheless, there are some limitations due to the cost of the enzymatic approach, the incomplete disruption of the cell wall and the complicated application in a commercial scale because of the different behaviour of the enzymes according to the environmental circumstances such as the amount of oxygen, the variety of nutrients and the operating temperature. This technique has never been applied to *S. repens* extraction.

#### Pressurized liquid/fluid extraction

Pressurized liquid extraction (PLE or PFE in case of a general fluid) is a novel and eco-friendly approach for the recovery of bioactives from plants. This method often requires water as the solvent and so it can keep away from the environmental and health risks due to the use of organic solvents. Operating at high temperature (till 200°C) and pressure (from 35 to 200 bar), PLE improves the quality of the extraction that can be carried out in a dynamic mode in which the solvent is incessantly pumped through the vessel, or in a static mode in which there are more cycles with a continuous replacement of the solvent [20]. At elevated temperatures there is a reduction of the viscosity of the solvent that can better penetrate the matrix extracting the analytes of interest, even if this approach cannot be used for the thermally unstable compounds and it could lead to a co-extraction of other compounds because of the decreased selectivity of extraction at higher temperatures. The elevated pressure permits maintaining the solvent in the liquid phase and the disruption of plant cells wall exerting pressure on the matrix. In place of organic solvents, it is possible to use additives like non-ionic surfactants, antioxidants like ascorbic acid, CO_2_ to drop aqueous pH or drying agents. Furthermore, PLE apparatus protects light sensitive and oxygen sensitive products from degradation, and it could be hyphenated with other modern extractive techniques like UAE to improve its efficacy.

### *Serenoa repens* extracts: therapeutic properties and implication for the treatment of BPH

Natural products recovered from plants and herbs have been using for centuries for the treatment of several pathologies including benign prostatic hyperplasia. Among the phytotherapeutics used for BHP, bioactives extracted from the fruit of the American dwarf palm *S. repens* is the most widespread thanks to its safety, tolerability profile, and clinical benefits. The composition of free fatty acids, methyl and ethyl esters, long chain esters and glycerides found in *S. repens* extracts (SrE) is different from a brand to another according to the extractive strategy used [21]. Although the specific mechanism of action of SrE has not been completely understood yet, it has been reported that the therapeutic agents could exert an inhibitory activity towards 5α-reductase, in addition to pro-apoptotic, anti-estrogenic and anti-inflammatory properties [22].

In the past, two reviews compared a large number of different brands of marketed *S. repens* extracts on the basis of the quali-quantitative composition in free fatty acids and their esterified forms [23] and on their corresponding inhibitory activity against the two isoforms of 5α-reductase [24,25]. Results were contrasting among these natural products (and above all among the batches of the same product) and, consequently, their clinical efficacy could be recognized different as well.

The database (Pubmed, Scifinder) investigation has been directed towards the most recent and relevant studies regarding the biological properties and therapeutic applications of *S. repens* extracts and the comparison among these commercial products. We chose “*Serenoa repens*” and “*Serenoa repens* extraction” terms for the bibliographic research. As a matter of this, we collected in Table 2 studies on different extracts and active principles of *S. repens* to demonstrate how, despite the pharmacological interest in this plant for the treatment of lower urinary tract symptoms, little is known about its phytochemical/pharmacological complexity and a better standardization could improve the interchangeability among these brand products.

**Table 2:** **Different extracts of different brands of *Serenoa repens *** **discussed and compared in this review**

**Extract (composition)**	**Extraction technique**	**Isolated active compound (%)**	**Ref.**
**Sabal Select** (>90% free fatty acids or their esterified forms, 0.01-0.15% fatty alcohols, 0.25-0.50% total sterols, 0.15-0.35% β-sitosterol)	Supercritical CO_2_	Oleic acid (15%)	[26]
		Lauric acid (15%)	
**Sabal Select** (>90% free fatty acids or their esterified forms, 0.01-0.15% fatty alcohols, 0.25-0.50% total sterols, 0.15-0.35% β-sitosterol)	Supercritical CO_2_	Lauric acid (30.2%)	[27,28]
		Oleic acid (28.5%)	
		Myristic acid (12.1%)	
		Palmitic acid (9.1%)	
		Linoleic acid (4.6%)	
		free fatty acids/mixed triglycerides ratio: ~55/45	
**Permixon**	Solvent (hexane) extraction	Oleic acid (36.0%),	[29-33]
Free saturated and unsaturated fatty acids (>90%)		Lauric acid (27.5%),	
		Myristic acid (12.0%)	
		Palmitic acid (9.7%).	
		Esterified FAs represent 7%, while the rest is composed of phytosterols, flavonoids, alcohols and polyprenic compounds	
**Prostasan**	Solvent (96% ethanol) extraction	Not reported	[34]
(95% total content of free fatty acids)			
**Prostasan**	Solvent (96% ethanol) extraction	Not reported	[35]
(86% total content of free fatty acids)			
**Saw Palmetto Berry Powder (SPBE)** (Madis Botanical, Inc., New Jersey)	Solvent (20% ethanol) extraction of (phyto)sterols	β-sitosterol,	[36]
		Stigmasterol,	
		Cholesterol	
(90% free fatty acids, alcohols and sterols)			
**PC-SPES**	Solvent (70% ethanol) extraction	Not reported	[37]
**Talso, Talso uno**	Supercritical CO_2_	acid lipophilic compounds, fatty alcohols and sterols as main components	[38]
**Prostamol Uno**	Not reported	Saturated and unsaturated fatty acids and phytosteroids	[39,40]
**ProstateEZE Max**	Not reported	*Serenoa repens* standardized to fatty acids, *Pygeum africanum* standardized to β-sitosterol, *Epilobium parviflorum*, *Cucurbita pepo* seed oil, lycopene	[41]
**Profluss**	Oily extract	*Serenoa repens* extract 85%, lycopene 6%, selenium	[42]
**SeR**	Solvent (ethanol) extraction	Not reported	[43]

From this synoptic summary it is possible to extrapolate that:

• As regards Sabalselect® by Indena (supercritical carbon dioxide extract, SFE), despite the high content in free fatty acids, the quantitative composition in its single components depends on the natural source variability. The major findings of this study are that lauric acid, oleic acid, myristic acid and linoleic acid, the major constituents of SFE, as well as SFE itself, actively bound to pharmacologically relevant (α1-adrenergic, muscarinic and 1,4-DHP) receptors in rat brain, and significantly inhibited 5α-reductase activity in rat liver. In addition, these components and the whole extract were shown to inhibit specific binding of [^3^H]prazosin in rat brain. Based on their IC_50_ values, the affinity for α1-adrenergic, 1,4-DHP, and muscarinic receptors displayed by linoleic acid, oleic acid, and myristic acid was 1.3-4.5 times greater than that of SFE. Conversely, the receptor binding activity of lauric acid and palmitic acid was similar to that of SFE. In general, the receptor binding activity of unsaturated fatty acids tended to be greater than that of saturated fatty acids (correlation of the pharmacological activity with the degree of unsaturation and bond length). SFE, lauric acid, oleic acid, myristic acid and linoleic acid inhibited 5α-reductase activity in a concentration-dependent manner. The inhibitory effect of each fatty acid was similar or slightly more potent than that of SPE. Consistent with these results, Raynaud *et al.* reported that palmitic acid was inactive in the inhibition of 5α-reductase [25]. The inhibitory effect on 5α-reductase activity by each fatty acid was roughly similar to their affinity for pharmacologically relevant receptors [26,27]. In a recent research, the effect on rat prostate gland contractility of this extract was evaluated after fractioning. The inhibition of prostatic smooth muscle contractions was preferentially induced by the ethyl acetate fraction (enriched in fatty acids) *via* a non-specific mechanism (not involving the inhibition of protein kinase C, myosin light chain kinase and Rho kinase) [28].

• Permixon® is the most studied extract in the literature both regarding its anti-proliferative activity and its anti-inflammatory ability. The former activity has been demonstrated, for the hexanic extract, against a large number of prostate carcinoma cell lines (PC3 and LNCaP) where it caused growth arrest and apoptosis at 50–80 μg/mL for 24–48 h (but not in MCF7 breast cancer cells). Treatment of cells with 100 μg/mL for 4 h resulted in morphological changes with massive vacuolization and swelling (derangement of the ultrastructure with major changes in membrane composition and function). Apoptosis seemed to depend on the intrinsic pathway, which appears to be triggered by opening of the mitochondrial PTP, a high-conductance channel that is involved in many forms of cell death, and complete release of mitochondrial TMRM (early mitochondrial depolarization and loss of membrane potential). Cell-cycle analysis showed the presence of a sub-G1 peak, a decreased G0/G1 peak and an accumulation of cells in G2/M phase after 48 h of treatment [29,30].

• With respect to the latter mechanism of action, different research groups highlighted the anti-inflammatory property of this plant. Multiple inflammation functional systems, such as Cytokines family, Glucocorticoid/PPAR signalling, MAPK signalling, TNF superfamily, and COX/LOX pathways, seemed to be modulated by LSESr treatment. Recently, Latil *et al*. [31] have studied how this biological property could be ascribed only to the hexanic extract of this plant and not to the supercritical CO_2_ extract. Both of the tested extracts belonged to six different batches of the same plant at Pierre Fabre Plantes et Industrie. Results showed that the hexanic extract was responsible of a strong attenuation of a large number of inflammation mediators and markers. Moreover, inhibition of MCP-1/CCL2 mRNA expression in a concentration-dependent manner has been registered only for the hexanic extract, whereas the supercritical CO_2_ extract was not endowed with this inhibitory effect. This is the only one example in the literature of a direct comparison of the different pharmacological profile associated to two extraction techniques showing the impact of this procedures on the quality/efficacy of phytotherapeutic products [31-33];

• Prostasan® has been also studied both for its anti-proliferative and anti-inflammatory activities (as an ethanolic extract). Comparison of the obtained results with respect to Permixon® led to similar results in terms of low cytotoxicity against normal cells, dose-dependent reduction of cellular growth (especially in AR-positive prostate and ER-positive breast cancer cells), apoptosis induction, inhibition of IL-12, MCP-1 and GM-CSF secretion and, consequently, decreased inflammatory processes and production of cytokine pro-inflammatory from macrophages. The content in free fatty acids is almost comparable to that of Permixon®. Moreover, inhibitory effects on the epithermal growth factor (EGF) and the Gram-negative bacteria cell wall component LPS-induced proliferation of prostatic cells PC-3 were observed with this extract. In the case of EGF, the mechanism involves competition for the EGF receptor. LPS exerts a milder but still significant induction on proliferation of prostate cells, supporting the role of bacterial infection in the origin of BPH. The mechanism behind *S. repens* extract effecting LPS actions on prostatic cells remains to be elucidated since it does not apparently affect Toll-like receptor 4 intracellular signalling [34,35];

• Saw Palmetto Berry Powder (SPBE) biological activity has been investigated with respect to its phytosterols content. With increasing concentration, the authors observed a reduction in cell growth compared to control cells in the following order of substance effectiveness: SPBE > β-sitosterol > stigmasterol, and an increase in cell growth with rising cholesterol concentration on prostate cancer cells (DU145). Analysis of cell cycle regulating proteins (p53, p27, p21) and 2D traction microscopy were also performed. p53 increased after treatment with this extract, whereas p27 and p21 decreased. This report justifies the administration of the entire phytocomplex to ensure a proper pharmacological efficacy not limiting the interest only to free fatty acids [36];

• PC-SPES is a combination of herbs containing flavonoids, alkaloids, polysaccharides, amino acids, and trace minerals. The 8 herbs used were chrysanthemum, isatis, licorice, *Ganoderma lucidum*, *Panax pseudo-ginseng*, *Rabdosia rubescens*, saw palmetto, and skullcap. PC-SPES mediated an anti-proliferative effect on prostate cancer cells (LNCaP, DU145, and PC3) *in vivo* and *in vitro*, induced apoptosis of LNCaP cells in a dose- and time-dependent manner and reduced prostate specific antigen (PSA) or AR levels in LNCaP cells and in more than 80% of individuals with prostate cancer. In addition in the same cell line, saw palmetto might inhibit cell growth down-regulating basal and DHT- or IL-6-induced PSA expression in cytoplasmic protein and AR expression in nuclear proteins. Previous studies have demonstrated that STAT3 signalling has a critical role in the tumour formation of prostate cancer and IL-6 treatment results in the activation of STAT3 in prostate cancer cells. This extract down-regulated the IL-6-induced level of the phosphorylated form of STAT3 in LNCaP cells blocking this pathway and markedly inhibiting the growth of LNCaP cells [37];

• The SG 291 extract (Talso, Talso uno) was analyzed by gas chromatography and investigated for its dual inhibitory influence on cyclooxygenase (COX, IC_50_ = 28.1 μg/mL) and 5-lipoxygenase (5-LOX, IC_50_ = 18 μg/mL). After alkaline hydrolysis, ether extraction and preparative TLC the SG 291 extract was separated in three fractions containing acid lipophilic compounds (A), fatty alcohols (B) and sterols (C). Only fraction A inhibited COX and 5-LOX as the native SG 291 extract, whereas the fractions B, C and β-sitosterol showed no inhibitory effect on both enzymes [38].

• Prostamol Uno (Berlin-Chemie AG/Menarini Group) was shown to possess anti-inflammatory and antiedematous effects reducing the proliferation of prostatic epithelium. Moreover, its pharmacological activity in rats with BPH was compared to that of a complex of peptides isolated from the cattle prostate. The results showed that both treatments reduced the acinar epithelial area in this experimental model, but the response to *S. repens* extract was characterized by an enhanced stromal/epithelial proportion [39]. In addition, the safety and tolerability of this extract have been evaluated in two dosage regimes in experimental animals [40].

• ProstateEZE Max (Caruso’s Natural Health) is an orally dosed herbal preparation containing *S. repens* (660 mg/day), among other prostatotropic agents of natural origin (*Cucurbita pepo*, *Epilobium parviflorum*, lycopene and *Pygeum africanum*). It has been studied in a short-term phase II randomized double-blind placebo controlled clinical trial to evaluate its efficacy and safety [41]. This combination of herbal extracts behaved as an effective treatment in the management of symptoms of BPH.

• Profluss® by Konpharma: to evaluate its efficacy on prostatic chronic inflammation, 168 subjects affected by LUTS due to bladder outlet obstruction were enrolled and subjected to Profluss with or without α-blockers treatment (“Flogosis And Profluss in Prostatic and Genital Disease” (FLOG) study). At follow-up there were statistical significant reductions of extension and grading of flogosis (mean values of CD20, CD3, CD68, and PSA). This product may have an anti-inflammatory activity that could be of interest in the treatment of PCI in BPH [42].

• SeR alcoholic extract, provided by Bernett, has been recently studied (alone and in association with selenium and lycopene) for the impact on the bax/bcl-2 ratio, caspase-3 activity, IAPs and survivin expression, and cytokines production in prostatic specimens from BPH patients. These analyses suggested that the impairment in the extrinsic pathway does not contribute to the cell death program in BPH, that the administration of SeR, either alone or in association, markedly mitigated survivin expression. Moreover, the expression of IL-6 was reduced preventing the histological features of BPH and inhibiting growth by 43.3% [43].

## Review and conclusions

Benign prostate hyperplasia is one of the most common diseases in male population. In addition to pharmacological therapy with α-blockers or 5α-reductase inhibitors, also bioactive compounds derived from plants play an important role in the treatment of BPH associated to LUTS. Despite the benefits obtained from SrE, the variety of the extractive techniques and strategies makes one extract different from another in terms of bioactives composition and this could affect the quality and the clinical effects of natural therapies of different brands even if derived from the same plant. Thus, the clinical and biological activities of one preparation cannot be extrapolated to other preparations of the same plant source. Moreover, standardization of the composition through alternative and more reproducible techniques is warranted due to the complex pharmacological profile of the whole phytocomplex of *S. repens*. Results from different clinical trials must be compared strictly according to the same validated extraction technique and/or content in active principles.

At this time, few studies made a comparison among these different extracts. The solvent (hexane) extraction of lipidosterolic composition from *S. repens* is the most investigated in clinical and experimental trials although further large comparative trials should explore the possible clinical influence of the different extractions processes.

## Competing interests

We disclose any conflict of interest such as consultancies, stock ownership or other equity interests, patents received and/or pending, or any commercial relationship which might be in any way considered related to the submitted article.

## Authors’ contributions

CDM conception and design, general supervision. SC conception and design, analysis and interpretation of data drafting of the manuscript. AG collection of data, drafting of the manuscript. GBDP conception and design, critical revision for intellectual content. CL general supervision, collection of data, CDN conception and design, general supervision, critical revision for intellectual content. All authors have made a significant contribution to the paper, and have read and approved the final draft. The work has not already been published and has not been submitted simultaneously to any other journal.

## Pre-publication history

The pre-publication history for this paper can be accessed here:

http://www.biomedcentral.com/1471-2490/14/63/prepub
